# “Flexible Assertive Community Mental Health Teams for the Treatment of Psychosis in Extraordinary Circumstances during Pandemics and Earthquakes 2020/2021: are we Flexible Enough?”

**DOI:** 10.1192/j.eurpsy.2022.188

**Published:** 2022-09-01

**Authors:** M. Rojnic Kuzman, S. Medved, S. Bjedov, M. Kovac, A. Mihaljevic-Peles

**Affiliations:** 1 University Hospital Center Zagreb, University of Zagreb, Department Of Psychiatry, Zagreb School Of Medicine, Zagreb, Croatia; 2 University Hospital Center Zagreb, Department Of Psychiatry And Psychological Medicine, Zagreb, Croatia; 3 Neuropsychiatric hospital Dr. Ivan Barbot, Psychiatry, Popovaca, Croatia

**Keywords:** Covid-19 pandemic, earthquake, Mental health services, community mental heath teams

## Abstract

During the ongoing COVID-19 pandemic, two regions in Croatia, Zagreb and Petrinja in the Sisak-Moslavina County experienced strong earthquakes, on the March 22^nd^ 2020 and on the 29th of December 2020. Both earthquakes, but especially the later one resulted on severe damage of the regions and caused severe disruptions in (mental) health service delivery. In these circumstances, the RECOVER-E project (LaRge-scalE implementation of COmmunity based mental health care for people with seVere and Enduring mental ill health in EuRopE), which was ongoing in Croatia from 2018-2022 aimed to implement flexible assertive community treatment as the new health care service for persons with severe mental illness (SMI). Since the pandemic and earthquakes have significantly influenced the life circumstances of all RECOVER-E project participants with SMI, we wanted further to examine the impact of health care delivery on mental health and the response to stress caused by a pandemic and earthquake in the patients with SMI involved in the project, in the first and second wave of the COVID-19 pandemics. Additionally, using case series, we will demonstrate the community mental health teams’ contributions in managing SMI after a double disaster in providing feasible, comprehensive, and accessible mental health services.1

**Disclosure:**

No significant relationships.

